# Revealing the Hypoglycemic Effects and Mechanism of GABA-Rich Germinated Adzuki Beans on T2DM Mice by Untargeted Serum Metabolomics

**DOI:** 10.3389/fnut.2021.791191

**Published:** 2021-12-14

**Authors:** Xiujie Jiang, Qingpeng Xu, Aiwu Zhang, Yong Liu, Zhijiang Li, Huacheng Tang, Dongmei Cao, Dongjie Zhang

**Affiliations:** ^1^National Coarse Cereals Engineering Research Center, Heilongjiang Bayi Agricultural University, Daqing, China; ^2^College of Food Science, Heilongjiang Bayi Agricultural University, Daqing, China; ^3^Experimental Equipment Management Center, Heilongjiang Bayi Agricultural University, Daqing, China

**Keywords:** GABA, germinated adzuki beans, type 2 diabetes, metabolomics, hypoglycemic effect

## Abstract

Type 2 diabetes mellitus (T2DM) is one of the most common metabolic diseases, and exploring strategies to prevent and treat diabetes has become extremely important. In recent decades the search for new therapeutic strategies for T2DM involving dietary interventions has attracted public attention. We established a diabetic mouse model by feeding mice a high-fat diet combined with injection of low-dose streptozotocin, intending to elucidate the effects and possible mechanisms of different dosages of γ-aminobutyric acid (GABA)-rich germinated adzuki beans on the treatment of diabetes in mice. The mice were treated for 6 weeks either with increasing doses of GABA-enriched germinated adzuki beans, with non-germinated adzuki beans, with GABA, or with the positive control drug metformin. Then, the blood glucose levels and blood lipid biochemical indicators of all the mice were measured. At the same time, serum differential metabolite interactions were explored by UPLC-Q/TOF-MS-based serum metabolomic analysis. The results showed that body weight and fasting blood glucose levels were significantly reduced (*P* < 0.05). We also report improved levels of total cholesterol, triglycerides, aspartate aminotransferase, alanine aminotransferase, urea, and serum creatinine. We observed a significant improvement in the homeostasis model assessment of the beta cell function and insulin resistance (HOMA-β and HOMA-IR) scores (*P* < 0.05) in the group of mice treated with the highest dose of GABA-enriched germinated adzuki beans. In addition, the metabolic profiles of the serum were analyzed, and 31 differential metabolites including amino acids and lipids were obtained. According to the Kyoto Encyclopedia of Genes and Genomes pathway enrichment analysis, this was found to be correlated with nine significantly enriched metabolic pathways involving the up-regulation of levels of L-serine, SM (d18:1/22:1(13Z)), L-histidine, creatine, and 3-indoleacetic acid. Our data suggest that the hypoglycemic effect of GABA-enriched germinated adzuki beans on diabetic mice may be related to improving tryptophan metabolism, glycerol phospholipid metabolism, sphingosline metabolism, and the glycine, serine, and threonine metabolic pathways. This study provides a reference for the application of GABA-enriched germinated foods in type 2 diabetes and could provide a cue for searching biomarkers to be adopted for T2DM diagnosis.

## Introduction

Type 2 diabetes mellitus (T2DM) is a common metabolic disease characterized by the dysregulation of glucose homeostasis. Traditional lifestyles and dietary habits have undergone fundamental changes, such as an increase in high-calorie food intake, excessive smoking, and alcohol consumption, and a reduction in physical exercise, leading to an increase in global medical expenditure (1, 2). Currently, the main strategies for treating T2DM are the adoption of a healthy diet with moderate but regular exercise, and, if strictly necessary, hypoglycemic drugs. A healthy diet is the cornerstone of diabetes treatment; therefore, investigating new dietary intervention therapeutic strategies for T2DM has become a significant research hotspot.

Legumes are key components of several plant-based diets and are widely consumed worldwide. Adzuki beans (*Vigna angularis*), one of the most ancient crops cultivated in China, are an excellent source of protein and non-digestible saccharides, have high nutritional value, and are a great source of dietary fiber, vitamins, and minerals (3). It is also sometimes advertised as one of the “weight-loss beans,” and its consumption is encouraged to control body weight, because of its high protein and low-fat content and a variety of bioactive substances (4). Polyphenols, flavonoids, and saponins from adzuki beans have been reported to possess many biological activities, such as anti-inflammatory (5), anti-obesity (6–8), and anti-cancer effects (9), with a wide range of potential health benefits.

Germination is a simple and inexpensive technique that causes changes in the nutritional and functional properties of germinated seeds. Germination can effectively activate endogenous enzyme activity in seeds and cause multiple physiological changes (10). Hence, it also increases the content of nutritional compounds such as polyphenols, ascorbic acid, thiamine, and especially, γ-aminobutyric acid (GABA) (11, 12). GABA is produced mainly from glutamate (Glu) *via* the catalyzation of Glu decarboxylase during germination (13). GABA is a non-essential amino acid that serves as a major neurotransmitter in the human nervous system and plays an important role in relieving anxiety, regulating blood pressure, and preventing cancers (14). Besides, a recent study discovered that GABA can induce α cell regeneration and promote α cell transformation into β-like cells *in vivo* (15) and could improve blood glucose and insulin levels, mainly through the downregulation of the glucagon receptor gene expression (16), which provides a new idea for the treatment and control of diabetes development.

Currently, a variety of GABA-enriched food products are developed by soaking and germination processing methods, to improve the sub-health status of modern people *via* the intake of GABA-enriched foods in daily life. For example, germinated rice bran (GRB) is a good source of GABA, and it has been reported that the regular consumption of GRB in the diet can attenuate insulin resistance and improve balance energy (17). GABA-enriched yogurts appear to be beneficial to islet cells and insulin sensitivity in a mouse model of T2DM (18). As adzuki beans are rich in glutamic acid, they could be used to produce nutritious, GABA-enriched, germinated foods, as it happens already with other grains, but very limited studies have been carried out on the health benefits of germinated adzuki beans for the prevention or treatment of T2DM. In particular, germinated adzuki beans belong to a multi-component substance. Therefore, it is of great significance to choose a scientific method to explore the mechanism of GABA-enriched germinated adzuki beans regulating blood glucose in T2DM mice.

In recent years, based on the UPLC-Q/TOF-MS metabonomics technology has been applied in the field of food and nutrition science due to its higher detection efficiency. It is helpful to effectively complete the qualitative and quantitative analysis of metabolites and can evaluate the overall changes of small molecule metabolites with a relative molecular weight of <1,000 in biological samples (19). To some extent, it provides a scientific basis for the pathophysiological pathway analysis of type 2 diabetes (20). Some reports have confirmed that triacylglycerols (TAGs) (21) and blood amino acids (22) may be associated with the risk of type 2 diabetes by metabolomic analysis.

For this reason, we set aimed to investigate the beneficial effects of different doses of GABA-enriched germinated adzuki beans on T2DM mice and were able to explain the anti-diabetes mechanism of GABA-enriched germinated adzuki beans through metabolomics. The objective of the present study was to reveal the hypoglycemic effect of dietary intervention with GABA-enriched germinated adzuki beans in type 2 diabetic mice and the metabolic mechanism of action.

## Materials and Methods

### Materials and Chemicals

Adzuki beans (*Vigna angularis*) were purchased from Daqing Linyuan Coarse Cereals Production Research Demonstration Base (Daqing, China) and stored in a refrigerator (4°C). GABA, streptozotocin (STZ), acetonitrile, and methanol (chromatography grade) were obtained from Sigma Co. Ltd. (St Louis, MO, USA). Sinocare Anwen+DZ model blood glucose test strips and blood glucose meters were purchased from Sinocare Ltd. Fasting insulin (FINS) test kits were purchased from Shanghai Enzyme-linked Biotechnology Co. Ltd. (Shanghai, China). The Health Check kit 16 biochemical reagent discs for measuring the total protein (TP), total bilirubin (TB), alanine aminotransferase (ALT), and aspartate aminotransferase (AST) contents were purchased from Seamaty Technology Co., Ltd. (Chengdu, China). High-fat feed [TP23300: 19% protein, 21% carbohydrate, 60% fat, heat density (5.1 kcal/g)] was purchased from TROPHIC Animal Feed High-Tech. Co., Ltd., Nantong, China. Basic feed [TP 23302: 19% protein, 71% carbohydrate, 10% fat, heat density (3.6 kcal/g)] was purchased from TROPHIC Animal Feed High-Tech. Co. Ltd. according to the national standard (GB 14924.3-2010). All other reagents were of analytical grade and were purchased from local companies.

### Preparation of GABA-Rich Germinated Adzuki Beans

Carefully selected, the seeds of adzuki beans (200 g per batch) were soaked in 1.0% sodium hypochlorite (NaClO) for 15 min, washed three times with distilled water, and then immersed in a soaking solution containing 0.4 mmol/L CaCl_2_ (pH 5) at 32 ± 2°C for 16 h. The samples were then placed in germination trays sprayed with an appropriate amount of 2 mmol/L sodium Glu, covered with four layers of wet gauze, transferred into a vacuum drying oven (BZF-30, Shanghai Boxun Medical Biological Instrument Corp., Shanghai, China), and incubated at 31°C and −0.1 MPa for 16 h. After vacuum-assisted treatment, the germinated adzuki beans were removed from the vacuum drying oven and placed in a constant temperature and humidity incubator (ZXMP-R1230, Shanghai Zhicheng Analysis Instrument Manufacturing Co., Ltd., Shanghai, China). Germination was performed at 31°C for 48 h, at a relative humidity of 95 ± 3%, with moisture supplied by a humidifier, during which the adzuki beans were sprayed with 2 mmol/L sodium Glu once every hour. The germinated adzuki beans were dried (DHG-9070A, Changzhou Putian Instrument Manufacturing Co., Ltd., Changzhou, China) at 45°C for 24 h to reach a final moisture content of 8–10%. The GABA content in the germinated adzuki beans was determined using the high-performance liquid chromatography (HPLC) (Agilent 1200, USA) system (11). The process optimization of GABA enrichment *via* germination and GABA content determination of germinated adzuki beans were reported in our latest article (23).

### Animals and Feeding Conditions

Six-week-old C57BL/6J male mice (*n* = 64, weighing 18 ± 2 g) were obtained from the Changchun Yisi Experimental Animal Technology Co. Ltd. (License No: SCXK(Ji)2018-0007). The mice were kept in a clean animal room, at the Biotechnology Center of Heilongjiang Bayi Agricultural University, at a controlled temperature (25 ± 1°C), with a relative humidity of 50 ± 5%. During their entire stay at the facility, it was made sure that the mice were provided a clean and comfortable living environment. Throughout the experiment, the mice could freely drink water and had free access to food. Before the experiment, they were subjected to adaptive feeding for 1 week. This study was approved by the Animal Experiment Committee of Heilongjiang Bayi Agricultural University (Daqing, China). All animal procedures were performed in accordance with the Heilongjiang Experimental Animal Management Regulations in China.

### Diabetes Model Construction

A type 2 diabetes mouse model was established by high-fat feeding combined with low-dose STZ injection, as previously described by Zhang (24) and Shao (25), with slight adjustments. Healthy mice were divided into a normal control group (*n* = 8) and a T2DM model group (*n* = 56). The mice in the model group were fed with a 60% high-fat diet (HFD) (TP23300) for 4 weeks to induce obesity and were then administered a single intraperitoneal injection of 50 mg/kg STZ (0.1 mol/L citrate buffer pH 4.5 was used to dissolve STZ). On the 3rd day after STZ injection, the fasting blood glucose (FBG) levels were measured in the T2DM model group after fasting for 12 h. When the FBG levels in the T2DM model group exceeded 11.1 mmol/L and the FBG measurements were relatively stable for 3 consecutive days, it could be concluded that type 2 diabetes had been successfully induced, and the mice could be used in subsequent experiments (26).

### Experimental Design

**Figure 1:**
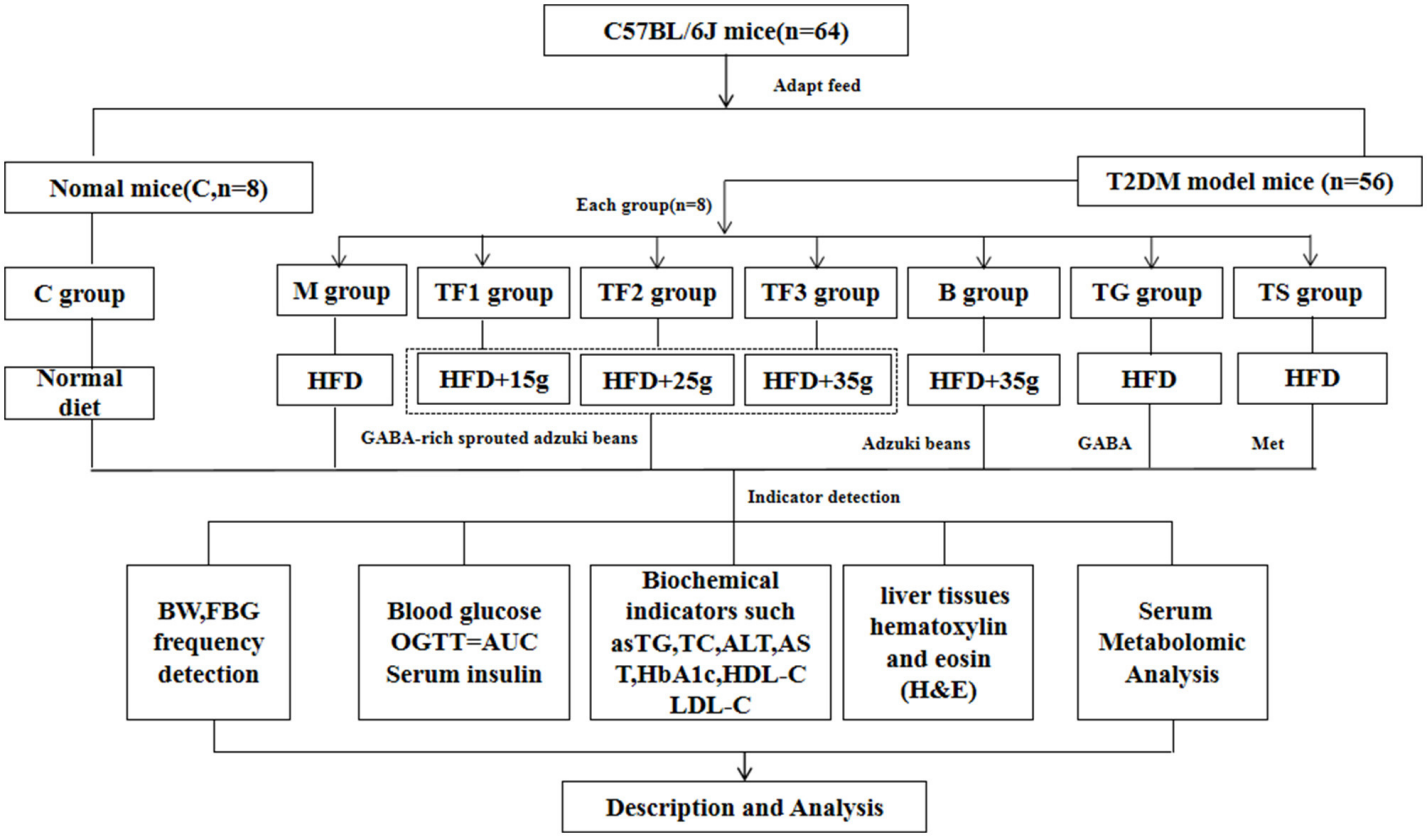
Schematic diagram of the animal experiment design.

Sixty-four healthy mice were randomly divided into two major groups (control group, *n* = 8; and T2DM group, *n* = 56) according to weight. Then, according to the FBG level in the T2DM model group, the mice were further randomly subdivided into seven groups, with each group consisting of eight mice (*n* = 8): the model group (M), the low-dose GABA-enriched sprouted adzuki beans group (15/100 g) (TF1), the medium-dose GABA-enriched sprouted adzuki beans group (25/100 g) (TF2), the high-dose GABA-enriched sprouted adzuki beans group (35/100 g) (TF3), the adzuki beans intervention group (35/100 g) (B), the GABA intervention (0.1 g/kg) (TG) group, and the metformin group (0.1 g/kg) (TS). The mice in the control group were fed a normal diet, the M, TG, and TS groups were fed high-fat feed (TP23300, 60% fat), and the TF1, TF2, TF3, and B groups were fed different doses of GABA-enriched germinated adzuki beans and adzuki beans replacement of some high-fat feed. All the mice were supplied with sufficient food and water and were given free access to food and water throughout each experiment until the end of the study. After 6 weeks of dietary and drug treatment, all the mice were anesthetized with pentobarbital sodium, blood samples were collected from the abdominal aorta, and the serum was separated under low-temperature conditions (4°C, 15 min) and then placed in an ultra-low temperature freezer (−80°C) until further analysis. After the liver, cecum, and colon tissues were removed, some were quickly frozen in liquid nitrogen, and a small part was fixed with 4% paraformaldehyde. The full experimental design is shown in Figure 1.

### Measurement of BW, FBG, HOMA-IR, and HOMA-β Levels

The FBG and body weight (BW) were measured weekly during the dietary and drug treatments. After deprivation of food for 12 h, the FBG values of all mice were measured using a Sinocare (Anwen+DZ model) blood glucose meter.

HOMA-IR and HOMA-β are indicators for evaluating mouse islet function in diabetic mice and were calculated using the following formulas:


(1) $$\begin{array}{c} \text{HOMA}-\text{IR}=\text{FINS}\times \text{FBG/22}.5 \end{array}$$

(2) $$\begin{array}{c} \text{HOMA}-\text{β }=(20\times \text{FINS)/(FBG}-3.5) \end{array}$$

### Biochemical Analysis

The FINS, FBG, triglyceride (TG), and total cholesterol (TC) were measured using reagent kits (Nanjing, Jiancheng, China), in accordance with the manufacturer's instructions. The serum levels of the ALT, AST, urea, and creatinine were measured using a biochemical analyzer (COBASc702, Roche, Switzerland).

### Serum Metabolomic Analysis

All frozen serum samples of the C, M, TF (high-dose GABA-enriched sprouted adzuki beans), TG, and TS groups were thawed at 4°C. Then, 100 μl aliquots of serum samples were transferred to 1.5 ml centrifuge tubes, and 400 μl of extraction solution, which had been prepared by adding equivalent amounts of methanol and acetonitrile, supplemented with 0.02 mg/ml of L-2-chlorophenyl alanine, was added. After vortexing for 30 s, the mixture was extracted ultrasonically at 5°C and 40 kHz for 30 min, and then the samples were placed at 20°C in a refrigerator for 30 min, centrifuged at 13,000 rpm at 4°C for 15 min, immediately collected and dried with nitrogen. One hundred microliters of reconstitution solution (acetonitrile:water = 1:1) were added to the dried pellet to dissolve it; the mixture was extracted using ultrasound at 5°C, 40 kHz, for 5 min and extracted again according to the above conditions, with the extraction time reduced to 5 min, and centrifuged for 10 min at 13,000 rpm and 4°C. Then, the supernatant was transferred to an autosampler vial, and 20 μl of the supernatant was taken as a quality control sample.

Serum metabolic profiling was performed using the UHPLC-Q-Exactive system (Thermo Fisher Scientific Technology Co., Ltd., China). The determination conditions were as follows: selected chromatographic columns, Acquity UPLC HSS T3 (100 × 2.1 mm, ID 1.8 μm), and could well balance polar and non-polar substances. Mobile phase A was 95% water + 5% acetonitrile, and mobile phase B was acetonitrile and isopropyl alcohol + 5% water. The injection quantity was 2 μl, the flow rate was 0.40 ml/min, and the column temperature was 40°C.

The mobile phases of A and B were run under the following optimized gradient program:

A:B (V:V = 100:0) at 0 min, A:B (V:V = 95:5) at 0.1 min, A:B (V:V = 75:25) at 2 min, A:B (V:V = 0:100) at 9 min A:B (V:V = 0:100) at 13 min, A:B (V:V = 100:0) at 13.1 min, and A:B (V:V = 100:0) at 16 min.

Quality control (QC) was prepared from a mixture of all samples of the extract, and the volume of each QC was the same as that of the sample, and the same method was used for the analysis. In the process of instrument analysis, after each 5–15 analytical samples, a QC sample is inserted to examine the stability of the entire detection process.

### Statistical Analysis

Descriptive statistics were analyzed using the SPSS 19.0 (IBM Corp, NY, USA) software, and the data are expressed as mean ± SD. ANOVA with Duncan's multiple range tests was performed to evaluate the data significance of general growth indexes and biochemical indicators [such as BW, FBG, insulin (INS), TG, TC, ALT, and AST] in the different groups. A probability level of *P* < 0.05 or 0.01 was considered statistically significant. The pacing chart and column chart were produced using Graph Pad Prism 7.0 (San Diego, CA, USA). Metabolic group data were analyzed and drawn on the cloud platforms of Shanghai Meiji Biomedical Technology Co. Ltd.

## Results

### Effect of Different Dosages of GABA-Enriched Germinated Adzuki Beans on Growth Performance and Glucose Homeostasis in Diabetic Mice

**Figure 2:**
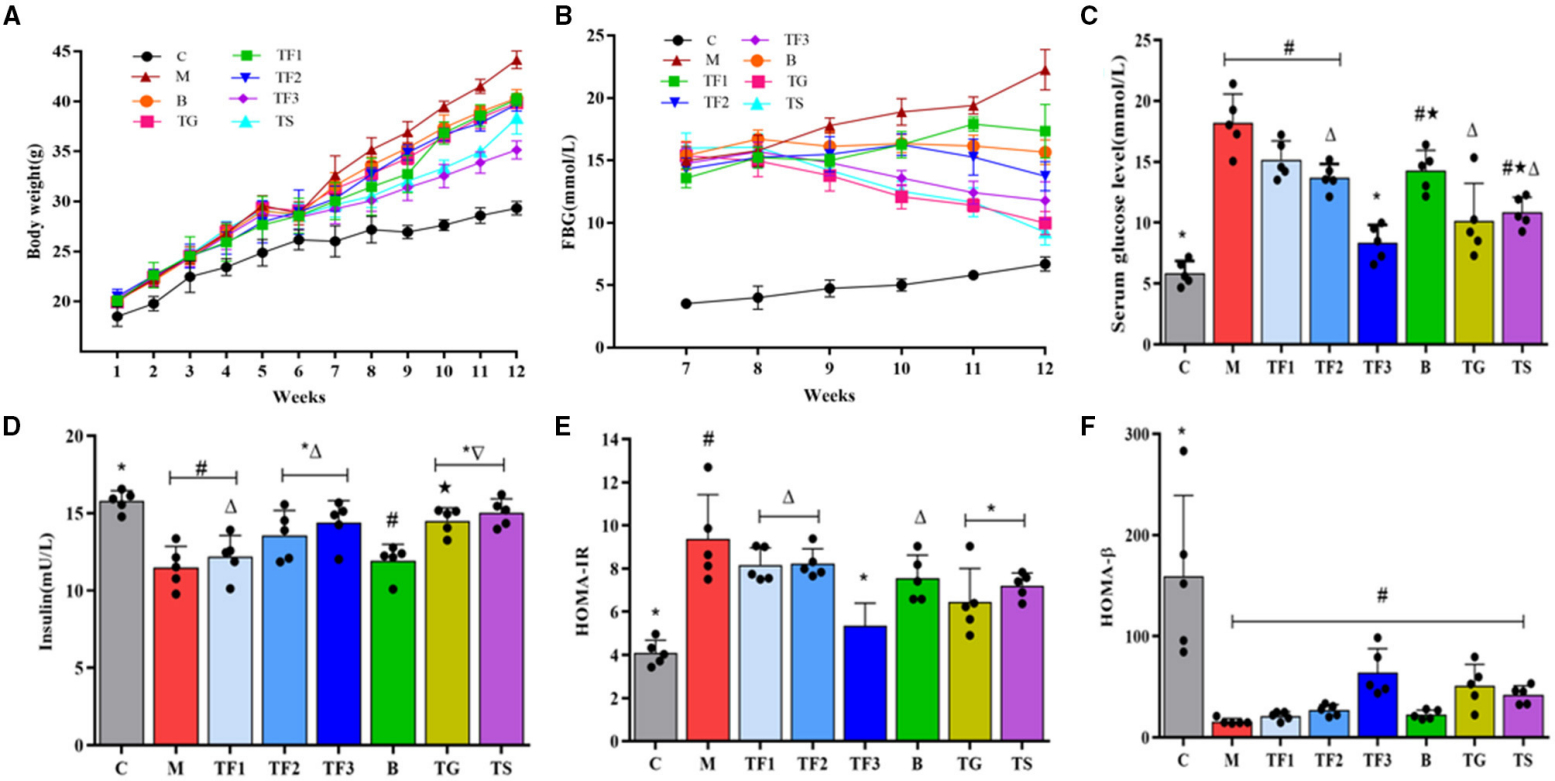
Effect of γ-aminobutyric acid **(GABA)**-enriched germinated adzuki beans on **(A)** body weight, BW, **(B)** fasting blood glucose, FBG, **(C)** serum glucose level, **(D)** insulin, INS, **(E)** insulin resistance **(HOMA-IR)**, and **(F)** beta cell function (HOMA-β) of diabetic mice after 12 weeks of feeding. C: control group; M: model group; TF1: low-dose GABA-enriched germinated adzuki beans treatment group (15 g/100 g); TF2: medium-dose GABA-enriched germinated adzuki beans treatment group (25 g/100 g); TF3: high-dose GABA-enriched germinated adzuki beans treatment group (35 g/100 g); B: adzuki beans intervention group (35 g/100 g); TG: GABA treatment group (0.1 g/kg); and TS: metformin treatment group (0.1 g/kg). Data are mean ± SD (*n* = 8 or 5). Different symbols indicate a significant difference according to ANOVA with Duncan's multiple range tests. (*P* < 0.05).

The effects of different dosages of GABA-enriched germinated adzuki beans on BW, FBG, serum glucose level, INS, HOMA-IR, and HOMA-β are shown in Figure 2. The BW and FBG of all experimental groups were measured weekly during the experiment (Figures 2A,B). As shown in Figure 2A, the BW of group M was markedly higher than that of group C (*P* < 0.05). The mice being fed with high-dose GABA-enriched germinated adzuki beans (TF3) and the mice undergoing metformin treatment (TS) had significantly lower BW than the mice in group M. In addition, the FBG level in the type 2 diabetes model group (M) was found to gradually increase in our experiment by feeding HFD, reaching up to 22.26 mmol/L by the end of the 12th week. Fortunately, the rapid increase in the FBG levels was inhibited by the treatment with different dosages of GABA-enriched germinated adzuki beans and metformin treatment. As shown in Figure 2B, after treatment with GABA-enriched germinated adzuki beans, compared with the FBG level in group M, the FBG levels of the mice in the TF1, TF2, and TF3 groups decreased by 22.01, 38.27, and 47.08%, respectively. This indicates that GABA-enriched germinated adzuki beans may have a certain benefit in balancing T2DM mouse hyperglycemia. At week 12, the serum glucose levels, INS, HOMA-IR, and HOMA-βwere measured in the mice of all groups. As shown in Figure 2C, the mice in group M had significantly higher values of serum glucose (18.21 mmol/L) than the mice in group C and TF3 (*P* < 0.05). The levels of insulin, HOMA-IR, and HOMA-β in group M were significantly different from those in group C (*P* < 0.05, Figures 2D–F). After 6 weeks of intervention, supplementation with different dosages of GABA-enriched germinated adzuki beans significantly improved insulin sensitivity compared with that of the diabetes model group (M), and group M had the worst sensitivity (Figure 2D). Compared with the HOMA-IR level of group C, the HOMA-IR levels of groups TF1, TF2, and B were significantly improved (*P* < 0.05), while those of TF3, TG, and TS were not significantly different (*P* > 0.05, Figure 2E). The HOMA-βlevels in group C were significantly higher than those in the other groups (*P* < 0.05). The HOMA-β level in the normal group mice had the highest value, compared with those of other groups; however, there was no significant difference in the HOMA-β level among groups TF1, TF2, TF3, and the model control (*P* > 0.05, Figure 2F). The results suggested that high-dose GABA-enriched germinated adzuki beans had beneficial effects on fighting increased weight and restoring glucose metabolism in diabetic mice.

### Effect of Different Dosages of GABA-Rich Germinated Adzuki Beans on Liver, Lipid, and Kidney Profile in Serum

**Figure 3:**
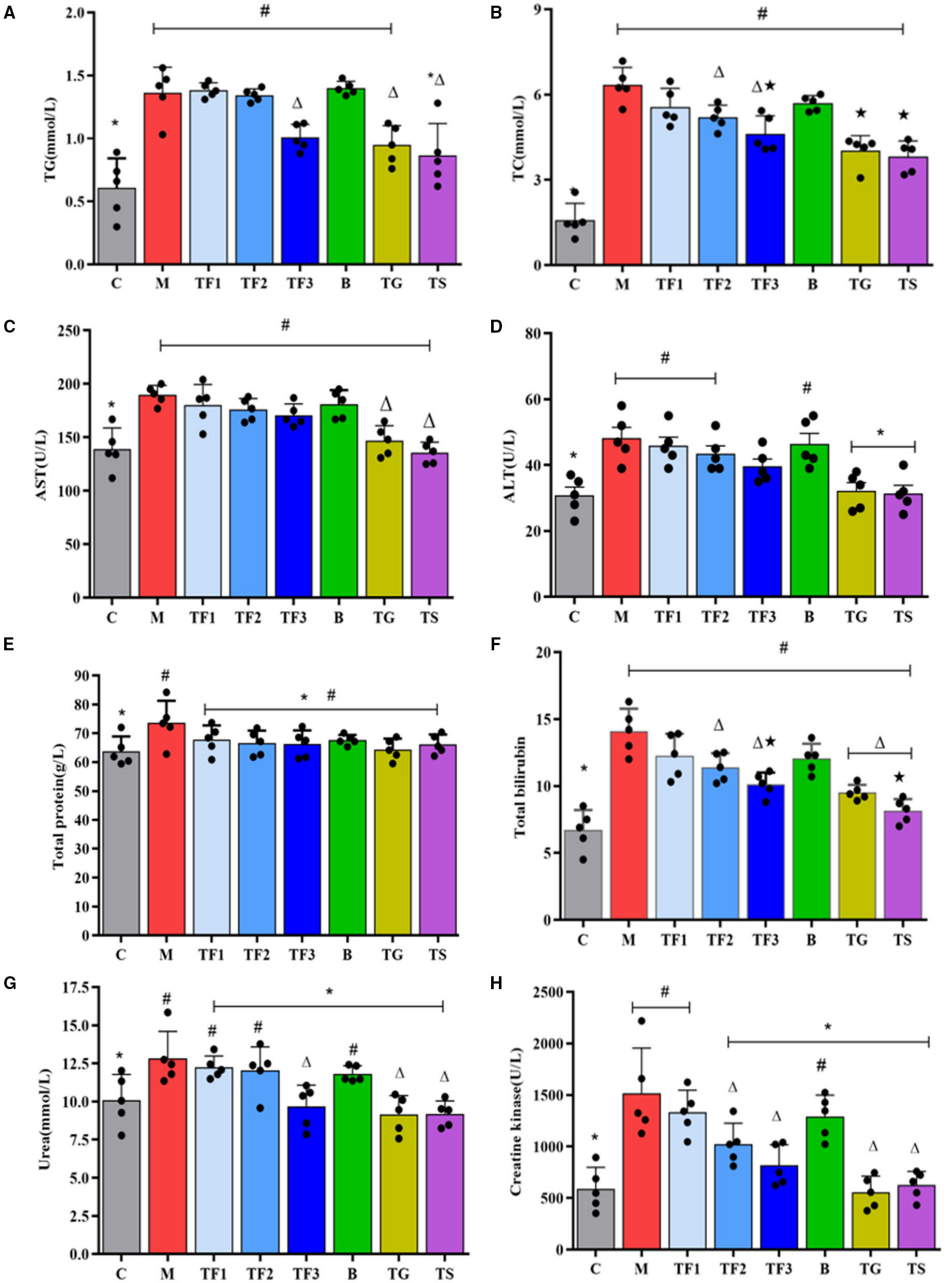
Effect of GABA-enriched germinated adzuki beans on biochemical functional indexes. **(A)** TG, **(B)** TC, **(C)** AST, **(D)** ALT, **(E)** total protein, **(F)** total bilirubin, **(G)** urea, and **(H)** creatine kinase of diabetic mice after 12 weeks of feeding. C: control group; M: model group; TF1: low-dose GABA-enriched germinated adzuki beans treatment group (15 g/100 g); TF2: medium-dose GABA-enriched germinated adzuki beans treatment group (25 g/100 g); TF3: high-dose GABA-enriched germinated adzuki beans treatment group (35 g/100 g); B: adzuki beans intervention group (35 g/100 g); TG: GABA treatment group (0.1 g/kg); and TS: metformin treatment group (0.1 g/kg). Data are mean ± SD (*n* = 8 or 5). Different symbols indicate a significant difference according to ANOVA with Duncan's multiple range tests. (*P* < 0.05).

Glycolipid metabolic disorders are an important causative agent of type 2 diabetes. The liver is the control center of blood glucose levels, as it plays an irreplaceable role in maintaining blood glucose homeostasis and regulating blood lipid concentrations (27). At the same time, the kidneys collaborate with the liver to maintain healthy circulation and metabolism. For this reason, lipid metabolism and liver and kidney functional indexes of the diabetic mice were assessed by measuring TG, TC, total protein (TP), total bilirubin (TB), ALT, AST, creatine kinase, and amylase levels. These results are shown in Figure 3. Compared with the levels of serum TG and TC in the control mice (group C), those in the diabetic model mice were significantly increased by feeding with HFD (*P* < 0.05), and after 6 weeks of treatment, the serum TG concentration in the TF3, TG, and TS groups was significantly reduced by 25.9, 30.2, and 36.4%, respectively (Figure 3A). TC was significantly reduced in group TF2 and TF3 (Figure 3B). The ALT and AST levels in the diabetic mice were significantly higher than those in normal mice, and after intervention with metformin (TS) and high-dose GABA-enriched germinated adzuki beans (TF3), there was no significant decrease in the AST level in mice (*P* > 0.05, Figure 3C); however, the ALT level in group TF3 was significantly lower than that in group M (*P* < 0.05, Figure 3D). Figures 3E,F shows the TP and TB in the serum of all mice. The levels of TP were improved in the model group (*P* < 0.05), but the administration of different dosages of GABA-enriched germinated adzuki beans and medication did not markedly decrease this indicator compared with that of the model group (*P* > 0.05, Figure 3E). The serum TB level of group M was 52.5% higher than that of group C; the TB level in group TF3 could fall by 28.3%, but the TS treatment group showed the highest decrease in TB levels (Figure 3F). The serum levels of urea and creatine kinase, which are indicators of kidney function, were significantly higher in group M than in group C (*P* < 0.05). After 12 weeks of treatment with high-dose GABA-enriched germinated adzuki beans (group TF3), with GABA (group TG), and with metformin (group TS), there was a significant decrease in the levels of urea and creatine kinase in all mice in these three groups (*P* > 0.05, Figures 3G,H).

### Serum Metabolomics of Diabetic Mice

#### Identification of Metabolites

According to the results of the multivariate statistical analysis of orthogonal partial least squares discriminant analysis (OPLS-DA), we determined a metabolite that significantly contributed to the group separation in the calculation of the VIP value and *P*. For VIP > 1 and *P* < 0.05, the compounds were considered to have statistical significance; subsequently, accurate molecular weight based on candidate differential metabolites (quality error within 10 ppm) was searched in online databases such as the human metabolome database (HMDB), METIN, and Kyoto Encyclopedia of Genes and Genomes (KEGG), and the obtained compound information and the tandem mass spectrometry (MS/MS) fragmentation spectrum provided the necessary structural information of the possible biomarkers.

**Figure 4:**
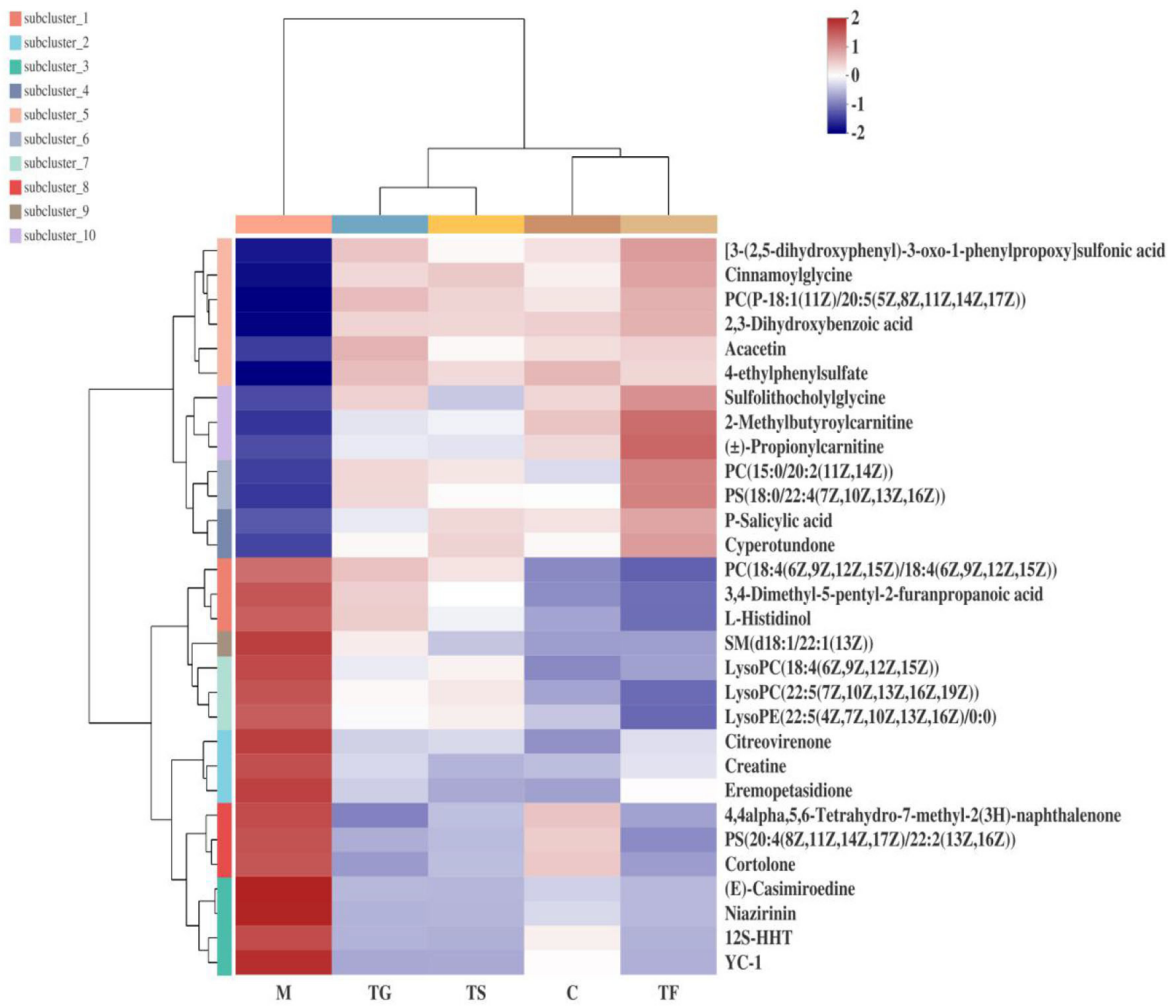
Heat map of different metabolites of type 2 diabetes mellitus (T2DM) mice after the intervention of GABA-enriched germinated adzuki beans. C: control group; M: model group; TF: high-dose GABA-enriched germinated adzuki beans treatment group (35 g/100 g); B: adzuki beans intervention group (35 g/100 g); TG: GABA treatment group (0.1 g/kg); and TS: metformin treatment group (0.1 g/kg). Each column in the figure indicates the average value of the packet sample (*n* = 6), and each line represents a metabolite in the ESI+ mode.

In the positive ion mode, 31 differential metabolites were identified as potential biomarkers in the normal group (C), model group (M), high-dose GABA-enriched germinated adzuki bean group (TF), GABA intervention group (TG), and metformin treatment group (TS). More complete information is provided in Table 1. Specifically, the levels of lysoPE (22:0/0:0) and PE (14:1(9Z)/22:1(13Z)), PC (15:0/20:2(11Z,14Z)), ()-propionylcarnitine, daidzein, PS(18:0/22:4(7Z,10Z,13Z,16Z)), ()-equol, phenol, and sulfolithocholylglycine showed significant reduction trends in the diabetic model group, while 12S-HHT, 3,4-dimethyl-5-pentyl-2-furanpropanoicacid, cytosine, eicosapentaenoic acid, 3-indoleacetic acid, niazirinin,1-methoxy-1H-indole-3-acetonitrile, isodomedin, L-serine, SM (d18:1/22:1(13Z)), ginsenosideC, YC-1, L-histidinol, and creatine showed significant increasing trends in the diabetic model group; after the high-dose GABA-enriched germinated adzuki bean dietary intervention, the levels of 12 metabolites that were significantly lower in T2DM mice were up-regulated, and the levels of 19 up-regulated differential metabolites were reduced, indicating that the high-dose GABA-enriched germinated adzuki bean dietary intervention caused the levels of these metabolites to be reversed by varying degrees. In order to analyze the changes in different metabolites more systematically, a heat map was constructed using the scipy (Python) software for the visualization of the 31 significantly differentially abundant metabolites (Figure 4). The use of the heat map allows a clearer and more pronounced representation of the distribution and the changes of the differentially expressed metabolites in the different treatment groups C, M, TF, TG, and TS. As shown in Figure 4, the control (C) group appears clearly separated from the model (M) group with significantly differential relative expressions of the metabolites. After administration of TF (high-dose GABA-enriched germinated adzuki beans), TG, and TS, the results showed that the TF and TS groups were clustered closer to the control (C) group. In other words, the differentially expressed metabolites in the TF and TS groups were similar to those in the C group, indicating that the dietary intervention of high-dose GABA-enriched germinated adzuki beans had a good adjustment effect on serum metabolites in T2DM mice.

**Table 1:** Analysis of common differential metabolites of ESI+ mode in the serum of mice from five group.

**No**.	**Metabolites**	***t*_*R*_ (min)**	**m/z**	**M vs. C**			**TF vs. M**			**TG vs. M**			**TS vs. M**		
				**VIP**	**FC**	**Trend**	**VIP**	**FC**	**Trend**	**VIP**	**FC**	**Trend**	**VIP**	**FC**	**Trend**
1	LysoPC(22:5(7Z,10Z, 13Z,16Z,19Z))	8.13	570.3554	1.78	1.09	↑	1.76	0.90	↓	1.50	1.95	↑	1.39	0.95	↑
2	12S-HHT	6.99	325.1746	2.24	1.37	↑	2.79	0.60	↓	3.25	0.60	↓	3.35	0.59	↓
3	LysoPE(22:0/0:0)	9.19	560.3681	1.03	0.96	↓	1.07	1.05	↑	1.03	1.03	↑	1.05	1.03	↑
4	PE(14:1(9Z)/22:1(13Z))	10.66	766.5359	1.63	0.91	↓	1.66	1.13	↑	1.48	1.08	↑	1.44	1.08	↑
5	3,4-Dimethyl-5-pentyl-2-furanpropanoic acid ()	5.64	221.1533	2.78	1.37	↑	2.63	0.70	↓	1.93	0.88	↓	2.36	0.83	↓
6	Cytosine	0.68	112.0507	1.18	1.06	↑	1.61	0.86	↓	1.43	0.92	↓	1.53	0.92	↓
7	PC(P-18:1(11Z)/20:5(5Z,8Z, 11Z, 14Z,17Z))	10.75	812.5556	1.80	0.89	↓	1.77	1.15	↑	2.03	1.14	↑	2.01	1.31	↑
8	PC(15:0/20:2(11Z,14Z))	11.21	794.5665	1.21	0.95	↓	1.61	1.11	↑	1.53	1.08	↑	1.45	1.07	↑
9	PC(18:4(6Z,9Z, 12Z,15Z)/18:4 (6Z,9Z,12Z,15Z))	9.84	796.4878	2.83	1.38	↑	2.76	0.69	↓	1.70	1.90	↑	2.02	0.87	↓
10	Eicosapentaenoic acid	7.82	303.2312	1.66	1.12	↑	2.46	0.75	↓	2.79	0.76	↓	2.76	0.78	↓
11	()-Propionylcarnitine	1.13	218.1384	1.63	0.91	↓	1.77	1.16	↑	1.33	1.07	↑	1.23	1.07	↑
12	3-Indoleacetic acid	1.26	176.0703	1.91	1.21	↑	2.48	0.67	↓	2.97	0.66	↓	2.96	0.68	↓
13	Niazirinin	1.30	339.1546	2.49	1.35	↑	2.35	0.71	↓	2.77	0.71	↓	2.86	0.71	↓
14	1-Methoxy-1H-indole-3-acetonitrile	1.61	187.0864	1.68	1.17	↑	2.37	0.68	↓	3.11	0.61	↓	3.18	0.63	↓
15	Daidzein	3.95	255.0647	2.36	0.66	↓	2.37	1.57	↑	2.70	1.56	↑	2.31	1.44	↑
16	Isodomedin	5.12	393.2242	2.57	1.45	↑	2.71	0.61	↓	2.93	0.66	↓	3.06	0.65	↓
17	LysoPC(18:4(6Z,9Z,12Z, 15Z))	7.31	538.2900	2.52	1.32	↑	2.11	0.77	↓	2.08	0.83	↓	1.91	0.85	↓
18	4-Hydroxy-17beta-estradiol-2-S-glutathione	7.37	626.2760	2.19	0.78	↓	1.85	1.24	↑	2.19	1.26	↑	2.17	1.23	↑
19	L-Serine	8.51	106.0500	1.48	1.19	↑	1.54	0.81	↓	1.99	0.76	↓	1.43	0.87	↓
20	SM(d18:1/22:1(13Z))	11.15	829.6171	2.36	1.30	↑	2.12	0.77	↓	1.85	0.86	↓	2.34	0.79	↓
21	PS(18:0/22:4(7Z,10Z, 13Z,16Z))	11.21	862.5533	1.51	0.92	↓	1.80	1.16	↑	1.73	1.11	↑	1.49	1.09	↑
22	PC(22:6(4Z,7Z,10Z, 13Z,16Z,19Z)/22:6(4Z, 7Z,10Z,13Z,16Z,19Z))	10.02	900.5499	1.18	0.94	↓	1.41	1.10	↑	1.41	1.08	↑	1.26	1.06	↑
23	PC(18:3(6Z,9Z,12Z)/ 20:5(5Z,8Z,11Z,14Z, 17Z))	10.02	824.5188	2.56	1.34	↑	1.85	0.85	↓	1.34	0.93	↓	1.77	0.89	↓
24	Ginsenoside C	9.93	848.5182	2.93	1.45	↑	2.57	0.69	↓	1.63	0.89	↓	1.82	0.88	↓
25	()-Equol	3.25	243.1013	2.65	0.66	↓	1.98	1.40	↑	2.74	1.51	↑	2.56	1.43	↑
26	YC-1	1.26	305.1280	3.60	2.38	↑	3.90	0.22	↓	4.61	0.21	↓	4.79	0.21	↓
27	L-Histidinol	0.66	124.0869	1.48	1.12	↑	1.51	0.87	↓	1.04	0.95	↓	1.37	0.92	↓
28	Creatine	0.62	132.0766	1.24	1.06	↑	1.00	0.95	↓	1.97	0.95	↓	1.40	0.94	↓
29	{[1-hydroxy-1-(1-oxo-1H-isochromen-3-yl)but-3-en-2-yl]oxy}sulfonic acid	0.52	313.0352	1.11	1.06	↑	1.01	0.95	↓	1.51	0.92	↓	1.61	0.91	↓
30	Phenol	2.25	95.0495	1.47	0.87	↓	1.65	1.20	↑	1.33	1.11	↑	1.46	1.13	↑
31	Sulfolithocholylglycine	4.69	496.2722	2.38	0.72	↓	2.68	1.52	↑	2.79	1.39	↑	1.88	1.21	↑

*↑ indicates the FC>1, ↓ indicates the FC <1*.

### Metabolic Pathway Analysis

**Figure 5:**
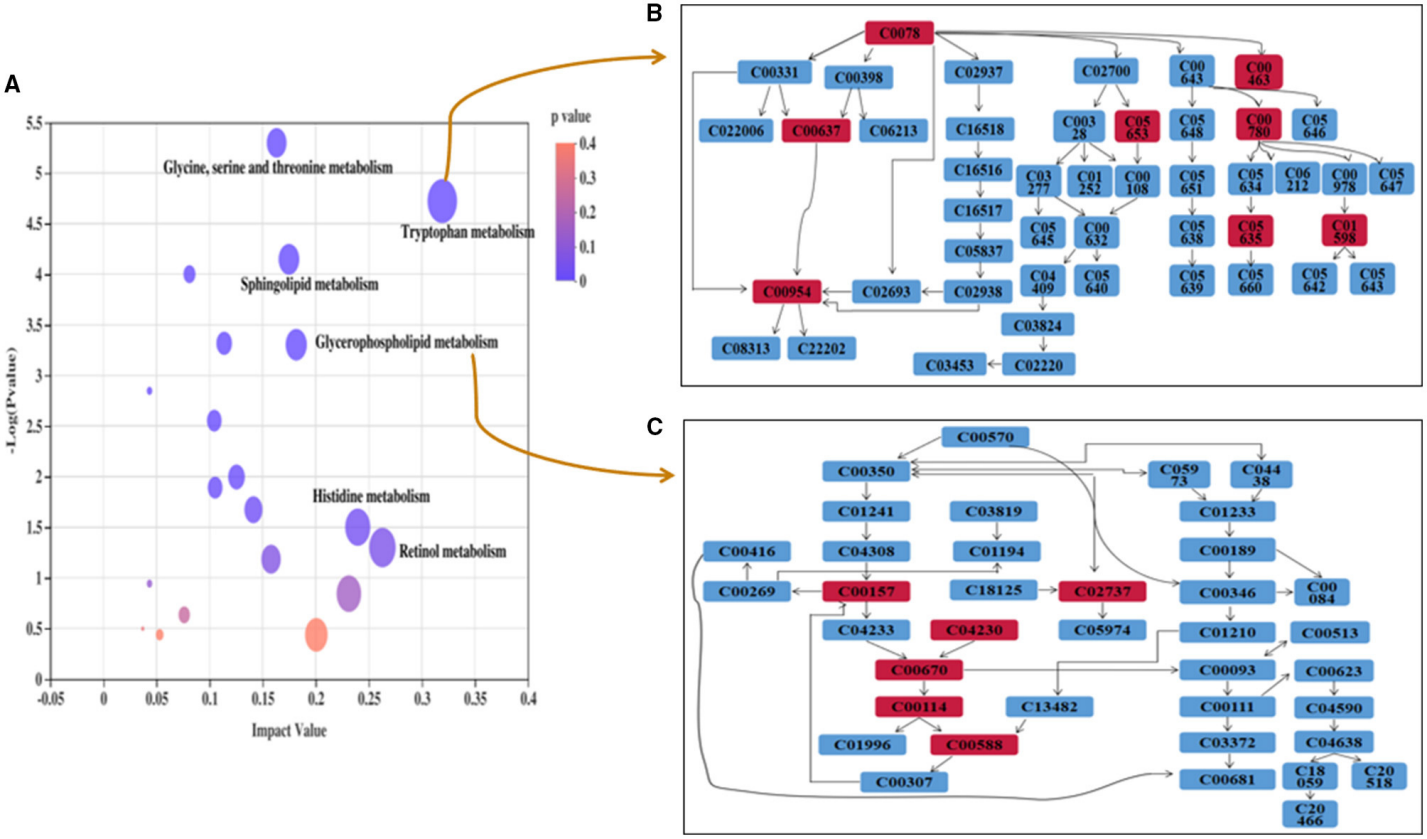
Metabolic pathway enrichment of differential metabolites in the serum of T2DM mice. **(A)** Each colored spot represents a metabolic pathway, such as (a) Tryptophan metabolism, (b) Glycerophospholipid metabolism, (c) Sphingolipid metabolism, (d) Glycine, serine, and threonine metabolism, (e) Ether lipid metabolism, (f) Steroid hormone biosynthesis. Color gradient represents the p-value size (orange: higher *p*-values and purple: lower *p*-values), and circle size represents the rank of pathway impact score (the larger the circle: higher impact score, and the smaller the circle: lower impact score). **(B)** Pathway of “Tryptophan metabolism.” **(C)** Pathway of “Glycerophospholipid metabolism.”

**Figure 6:**
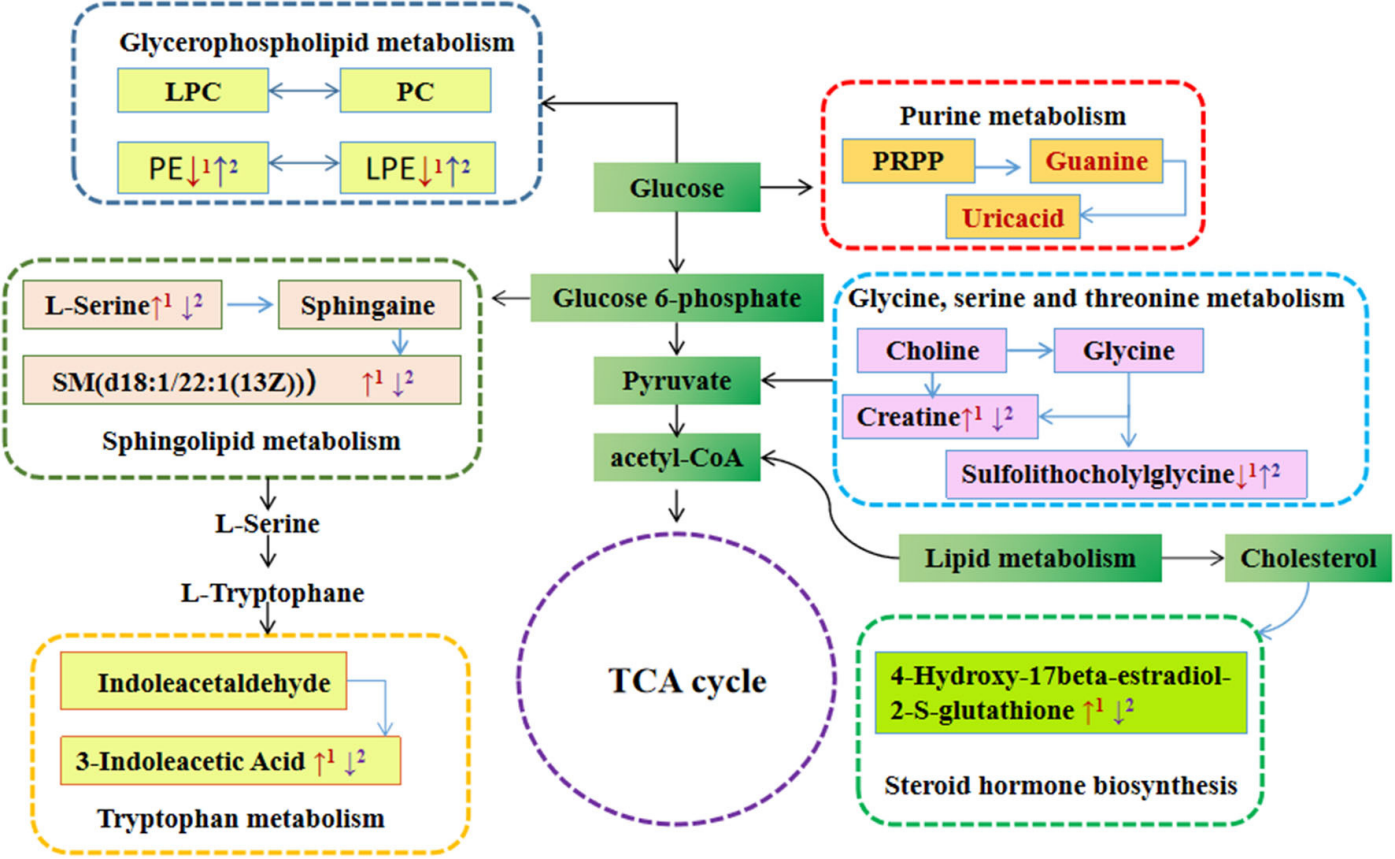
The map of pathway analysis of serum metabolism network. The red arrow indicates differential metabolites that are increased or decreased in the sera of mice in the M group compared with those in the C group. The purple arrow indicates differential metabolites that are increased or decreased in the sera of mice in the TF group compared with those in the M group. C: control group; M: model group; TF: high-dose GABA-enriched germinated adzuki beans treatment group (35 g/100 g).

To evaluate the effects of GABA-enriched germinated adzuki beans on the serum metabolism of diabetic mice, we carried out a metabolic pathway analysis of potential metabolites. Our analysis revealed significant differences in the phospholipid, amino acid, and steroid hormone concentrations. We comprehensively applied the online databases of KEGG and HMDB to explore the most relevant metabolic pathways and functions and further investigated the anti-diabetic mechanism of GABA-enriched germinated adzuki beans in the treatment of T2DM mice. At the same time, based on the path topology analysis of the influence factor (Impact ≥ 0.01) and *P*-values (*P* < 0.05), we screened the key metabolic pathways and finally obtained nine different metabolic pathways. These pathways were considered the most relevant pathways involved in T2DM. As shown in Figure 5 and Table 2, to be specific, the metabolites might be involved in tryptophan metabolism, glycerophospholipid metabolism, sphingolipid metabolism, glycine, serine, and threonine metabolism, ether lipid metabolism, steroid hormone biosynthesis, phenylalanine metabolism, aminoacyl-tRNA biosynthesis, and purine metabolism pathways, and the impact values of those pathways were 0.32, 0.18, 0.17, 0.16, 0.13, 0.11, 0.10, 0.08, and 0.04, respectively. Among them, tryptophan metabolism, glycerophospholipid metabolism, sphingolipid metabolism, and glycine, serine, and threonine metabolism had the highest impact values. Therefore, these four pathways were considered as the key metabolic enrichment pathways. Based on the KEGG database, we drew a map of these metabolic pathways to illustrate at which stages the GABA-enriched germinated adzuki bean dietary treatment could contribute to regulating blood glucose levels, as shown in Figure 6. Our results indicate that high doses of GABA-enriched germinated adzuki beans may be able to regulate blood glucose levels owing to their involvement in these four metabolic pathways.

**Table 2:** Statistical analysis results of the main metabolites in serum.

**No**.	**Pathway**	**Total**	**Matched number**	**Impact-value**	***P*-value-corrected**
1	Tryptophan metabolism	56	8	0.319275262	0.000303246
2	Glycerophospholipid metabolism	48	6	0.181670505	0.003412563
3	Sphingolipid metabolism	21	5	0.174796748	0.000855472
4	Glycine, serine and threonine metabolism	47	8	0.163188609	0.000120784
5	Ether lipid metabolism	21	3	0.12539185	0.04411498
6	Steroid hormone biosynthesis	89	8	0.113774364	0.003856628
7	Phenylalanine metabolism	46	5	0.10445548	0.014956558
8	Aminoacyl-tRNA biosynthesis	52	7	0.081081081	0.000959506
9	Purine metabolism	81	7	0.04341813	0.008539757

## Discussion

In our previous paper (23), we described how to achieve GABA enrichment in sprouted adzuki beans with our new optimized method of germination by vacuum and monosodium Glu treatment. The present study focused on the anti-diabetic activity of GABA-enriched germinated adzuki beans. We established a type 2 diabetic mouse model using HFD + STZ and supplemented the diets of the animals with GABA-enriched germinated adzuki beans to investigate the role of GABA-enriched germinated adzuki beans in preventing and managing diabetic conditions.

In the current study, the BW of the experimental mice in all groups exhibited different degrees of increase in the experiment (Figure 2A), whereas high-dose GABA-enriched germinated adzuki beans (TF3) and metformin (TS) treatments significantly reduced mice BW when compared with that of the model group. The results revealed that our treatments reduced the BW of the mice, especially for the high-dose GABA-enriched germinated adzuki bean-treated groups, regardless of the treatment strategy. Throughout the study, the TF3, TG, and TS groups exhibited significantly lower FBG than that in the model group after 10, 11, and 12 weeks of treatment (*P* < 0.05, Figure 2B). At the same time, the diabetic mice exhibited significantly higher FBG levels (Figure 2C), lower insulin sensitivity (Figure 2D), HOMA-β level (Figure 2F), and HOMA-IR level (Figure 2E) than those of the control group mouse, and other typical characteristics of T2DM; however, these symptoms were alleviated by the dietary intervention (TF2, TF3, B groups) and medication (TG and TS groups) in type 2 diabetes mice. Alternatively, there were no significant differences in the FBG, HOMA-IR, and HOMA-β indexes between the B and TF2 groups, but significant differences were observed between the B and TF3 groups (*P* < 0.05). In addition, the mice in group B, which had received the same dose of non-germinated adzuki beans, showed a poorer control of FBG than the mice in group TF3, which had been fed the highest dose of GABA-enriched germinated adzuki beans. These results implied that the hypoglycemic effect of TF3 was not due to the extra adzuki bean supplementation but the intrinsic activity of GABA in the germinated adzuki beans, and previous studies have also shown that the increased GABA content in germinated brown rice (28) and sprouted black rice (29) possibly played a significant role in the anti-diabetic properties attributed to these two cereal grains.

The liver plays an important role in achieving glucose homeostasis during diabetes (30). For this reason, we measured several biochemical indicators to evaluate the mouse hepatic health status (31). The liver is an essential control hub for glucose and lipid metabolism; serum levels of TG, TC, TP, TB, ALT, and AST provide essential information to judge liver functionality with regards to synthesis, bilirubin metabolism, and substance damage. For this reason, we compared biochemical parameters (such as TG, TC, ALT, and AST) of liver function between the control mice and the diabetic mice, and we observed that the diabetic mice exhibited signs of lipid metabolism disorder, which, if exacerbated, could generate adverse effects on liver tissue. Interestingly, the administration of a high-dose of GABA-enriched germinated adzuki bean to the mice in group TF3 remarkably reduced the TC and TG levels (*p* < 0.05, Figures 3A,B), while the serum levels of ALT, AST, TP, and TB also showed gradually declining trends (Figures 3C–F), indicating that the diet administered to the TF3 group had a great protective effect on the liver of the diabetic mice, which may be attributed to a large increase in GABA content in the germinated adzuki beans. Previous studies have confirmed that GABA has anti-diabetic effects, which are mainly achieved by inhibiting obesity-related inflammation and upregulating Treg responses *in vivo* (32), and it was recently shown that GABA intervention can effectively protect mice from acute liver injury *via* GABA-mediated STAT3 signaling (33). Additionally, in all HFD + STZ-treated T2DM mice, the blood urea and serum creatinine levels decreased in a dose-dependent manner with different dosages of GABA-enriched germinated adzuki bean treatment for 6 weeks (Figures 3G,H), which might be associated with reduced serum lipid profiles. Additionally, a complex connection between GABA signaling and amino acid metabolism disorders has been revealed (34).

To elucidate the anti-diabetes mechanism of GABA-enriched germinated adzuki beans for the potential treatment of T2DM, serum metabolite differences among the mice of groups C, M, TF, TG, and TS were analyzed with the help of metabolomics. Eventually, according to the variations of differential metabolites among groups, we identified 31 differential metabolites as potential biomarkers. It has been reported that the levels of lipid metabolites are high in individuals with type 2 diabetes (35). In this study, the levels of PCs [LysoPC (22:5(7Z,10Z,13Z,16Z,19Z)), LysoPC (18:4(6Z,9Z,12Z,15Z))PC(18:4(6Z,9Z,12Z,15Z)/18:4(6Z,9Z,12Z, 15Z)),PC (18:3(6Z,9Z,12Z)/20:5(5Z,8Z,11Z,14Z,17Z))] generally significantly increased in the diabetes model group (Table 1), and after treatment with high-dose GABA-enriched germinated adzuki beans, the levels of these metabolites were significantly increased, indicating that the lipid metabolic disorder in diabetic mice was improved, and the target metabolites were related to glycerophospholipid metabolism. Sphingolipid is a kind of amphoteric lipid containing a sphingosine skeleton. It has a variety of structures and belongs to a common lipid (36). It plays a key role in protecting the cell membrane function and regulating cell growth and apoptosis (37). Interestingly, we also identified two metabolites participating in the sphingolipid metabolism, namely, L-Serine and SM(d18:1/22:1(13Z)). Meanwhile, there were significantly decreased concentrations of L-Serine and SM(d18:1/22:1(13Z)) in the TF group compared with the M group and indicated that the degradation of the concentration of sphingolipid metabolites might have a positive role in anti-diabetes. Because sphingolipid decomposition metabolism might stimulate the effect of insulin signal transmission (38). In addition, Ye J et al. have found that serum sphingolipids of SM/Cer and SM d18:0/26:0 associated with insulin sensitivity (39), and serum sphingolipids of hexose ceramide were determined the significant correlation with T2DM (40). Furthermore, tryptophan, an essential nutrient in mammals, has a variety of functions, such as maintaining immune homeostasis (41). Presumably, endogenous metabolites are not only involved in the inhibition of oxidative stress and inflammation but also reflect the body's immune status (42). The present study found that serum tryptophan levels were decreased in mice with (HFD + STZ)-induced diabetes and that the levels of the tryptophan metabolite 3-indoleacetic acid were relatively higher in group M than in group C, while 3-indoleacetic acid levels were depleted in group TF compared to that in group M, indicating that the metabolism of tryptophan is accelerated in HFD+STZ diabetic mice. Matsuoka et al. revealed that tryptophan metabolism was more active in patients with diabetes than in healthy patients (43), which is consistent with our study, and the impact value of tryptophan metabolism was the largest in the significantly differentially altered metabolite pathway (nine types) according to MetPA analysis; therefore, it could be speculated that the diet supplemented with a high dose of GABA-enriched germinated adzuki beans had beneficial therapeutic effects on T2DM *via* tryptophan metabolism and its intermediates.

Another interesting finding in our metabolomic profiling of diabetic mice was the dysregulation of glycine, serine, and threonine metabolism pathways; the results of this study showed that the creatine metabolite content in the model group was significantly higher than that in the control group. In contrast, after the GABA-enriched germinated adzuki bean dietary intervention, the content of creatine metabolites in the TF group was lower than that in the model group. This indicated that the relative concentrations of glycine, serine, and threonine metabolism intermediates were up-regulated, and their concentrations were positively correlated with T2DM progression. In our research, the high dose GABA-enriched germinated adzuki beans (TF) could downregulate the L-histidine and L-serine levels, which is conducive to accelerating the citric acid cycle and generating more alpha-ketoglutarate, eventually downregulating the blood glucose level of the diabetic mice. Overall, these results reveal that the disturbance of metabolites in mice with HFD+STZ-induced diabetes may involve complex biological processes, depending on the severity of the disease.

## Conclusion

In summary, this study has demonstrated the anti-diabetic property of different dosages of GABA-rich germinated adzuki beans using the HFD+STZ-induced diabetic mice model, especially in diabetic mice receiving dietary intervention of the high-dose GABA-rich germinated adzuki beans could decrease the FBG level, improve insulin sensitivity, and recover the lipid, liver and kidney indicators (TC, TG, AST, ALT, Ure, and Serum creatinine)to various degrees in sera of diabetic mice.

Based on the untargeted metabolomics approach, we aimed to further analyze the mechanism of GABA-rich germinated adzuki beans regulated the blood glucose at the metabolite level. Interestingly, 31 differential metabolites were identified as potential biomarkers, including amino acids and lipids. We speculated that the dietary intervention of GABA-rich germinated adzuki beans had therapeutic effects mainly through regulating tryptophan metabolism, glycerophospholipid metabolism, sphingolipid metabolism, glycine, serine and threonine metabolism, ether lipid metabolism, steroid hormone biosynthesis, phenylalanine metabolism, aminoacyl-tRNA biosynthesis, and purine metabolism. Notably, tryptophan metabolism is the most important in the significantly differential metabolites changed pathway and may be a potential target for future diabetes treatment.

## Data Availability Statement

The original contributions presented in the study are included in the article/Supplementary Material, further inquiries can be directed to the corresponding author.

## Ethics Statement

The animal study was reviewed and approved by Animal Experiment Committee of Heilongjiang Bayi Agricultural University (Daqing, China).

## Author Contributions

XJ was responsible for conceiving and designing the experiments and drafted the manuscript. QX performed the metabolomics experiment and helped data analysis. AZ was responsible for supervising the animal experiment and experimental activity. YL and ZL collected information materials and conceptual treatments. HT and DC interpreted the biochemical pathways and helped in perfecting the manuscript. DZ handled the supervision throughout research and manuscript publishing. All authors have read and approved the final manuscript.

## Funding

This work was supported by the cooperation research and application demonstration of the refined processing key technology of coarse cereal foods (2018YFE0206300) and the Research Team Project of the Natural Science Foundation of Heilongjiang Province, China (Grant No. TD2020C003).

## Conflict of Interest

The authors declare that the research was conducted in the absence of any commercial or financial relationships that could be construed as a potential conflict of interest.

## Publisher's Note

All claims expressed in this article are solely those of the authors and do not necessarily represent those of their affiliated organizations, or those of the publisher, the editors and the reviewers. Any product that may be evaluated in this article, or claim that may be made by its manufacturer, is not guaranteed or endorsed by the publisher.
